# Breaking Diagnostic Barriers: Vision Transformers Redefine Monkeypox Detection

**DOI:** 10.3390/diagnostics15131698

**Published:** 2025-07-03

**Authors:** Gelan Ayana, Beshatu Debela Wako, So-yun Park, Jude Kong, Sahng Min Han, Soon-Do Yoon, Se-woon Choe

**Affiliations:** 1 Department of Biomedical Engineering, Kumoh National Institute of Technology, Gumi 39253, Republic of Korea; gelan.zewdie@utoronto.ca (G.A.);; 2 School of Biomedical Engineering, Jimma Institute of Technology, Jimma University, Jimma 378, Ethiopia; 3 Artificial Intelligence & Mathematical Modeling Lab (AIMM Lab), Dalla Lana School of Public Health, University of Toronto, 155 College St Room 500, Toronto, ON M5T 3M7, Canada; 4 Center of Biomedical Engineering, Jimma University Medical Center, Jimma 378, Ethiopia; 5 Department of Computer Science, Gwinnett Technical College, Lawrenceville, GA 30043, USA; 6 Department of Chemical and Biomolecular Engineering, Chonnam National University, Yeosu 59626, Republic of Korea; 7 Emerging Pathogens Institute, University of Florida, Gainesville, FL 32608, USA

**Keywords:** Mpox, vision transformer, detection

## Abstract

**Background/Objective:** The global spread of Monkeypox (Mpox) has highlighted the urgent need for rapid, accurate diagnostic tools. Traditional methods like polymerase chain reaction (PCR) are resource-intensive, while skin image-based detection offers a promising alternative. This study evaluates the effectiveness of vision transformers (ViTs) for automated Mpox detection. **Methods:** By fine-tuning a pre-trained ViT model on an Mpox lesion image dataset, a robust ViT-based transfer learning (TL) model was created. Performance was assessed relative to convolutional neural network (CNN)-based TL models and ViT models trained from scratch across key metrics: accuracy, precision, recall, F1-score, and area under the receiver operating characteristic curve (AUC). Furthermore, a transferability measure was utilized to assess the effectiveness of feature transfer to Mpox images. **Results:** The results show that the ViT model outperformed a CNN, achieving an AUC of 0.948 and an accuracy of 0.942 with a *p*-value of less than 0.05 across all metrics, highlighting its potential for accurate and scalable Mpox detection. Moreover, the ViT models yielded a better hypothesis margin-based transferability measure, highlighting its effectiveness in transferring useful learning weights to Mpox images. Gradient-weighted Class Activation Mapping (Grad-CAM) visualizations also confirmed that the ViT model attends to clinically relevant features, supporting its interpretability and reliability for diagnostic use. **Conclusions:** The results from this study suggest that ViT offers superior accuracy, making it a valuable tool for Mpox early detection in field settings, especially where conventional diagnostics are limited. This approach could support faster outbreak response and improved resource allocation in public health systems.

## 1. Introduction

Monkeypox (Mpox) is a viral zoonotic disease that has become a global health threat [1]. The disease, caused by the Mpox virus, is transmitted from animals to humans and, in some cases, from human to human. Symptoms include fever, rash, and swollen lymph nodes, with complications that can lead to serious health consequences, particularly in immunocompromised individuals [2]. The resurgence of Mpox in non-endemic regions underscores the critical need for rapid, accurate, and accessible diagnostic tools [3].

Currently, the gold standard for Mpox diagnosis is polymerase chain reaction (PCR), which detects the viral deoxyribonucleic acid (DNA) with high specificity [4]. However, PCR tests require laboratory infrastructure, skilled personnel, and time, making them less practical for comprehensive and rapid screening in resource-limited settings [4,5]. Imaging techniques, such as dermatoscopy and digital imaging, have been investigated as supplementary diagnostic methods [6]. These techniques allow for non-invasive and rapid assessments, but their effectiveness largely depends on the experience of the clinician and the quality of the image analysis tools used [7].

Artificial intelligence (AI) has emerged as a powerful tool for analyzing Mpox images, enabling significant advancements in its diagnosis and management [8]. However, its use also raises critical concerns, particularly around data privacy and ethical considerations [9]. These include the risk of compromising patient confidentiality and the potential for biases within AI algorithms to impact diagnostic outcome. Nevertheless, deep learning algorithms have been successfully applied to analyze images of Mpox-related skin lesions and achieved high detection and classification accuracy. Deep learning algorithms have been successfully applied to analyze images of Mpox-related skin lesions and achieved high detection and classification accuracy [10]. These AI-driven models have consistently outperformed conventional diagnostic methods in identifying intricate patterns that are critical for early and precise diagnosis [11]. Researchers have used different dataset sizes to train these models, resulting in systems that provide reliable and consistent assessments [12]. Convolutional neural networks (CNNs) have been used extensively for tasks such as detection, classification, and anomaly detection [13,14]. CNNs excel at capturing local patterns in images through convolutional layers but often struggle with global context and long-range dependencies, which are critical in complex visual tasks [15,16]. Vision transformers (ViTs) have recently emerged as a powerful alternative to CNNs [17,18,19,20,21]. However, the large parameter size of a ViT hinder its application to small number datasets, as it needs sophisticated training and deployment capability that has promoted the creation of new methods [22]. For instance, Li et al. [23], proposed plain-backbone detection that largely maintains the independence of the general-purpose backbones and the downstream task-specific designs during transfer learning using ViTs. Moreover, a deep multimodal representation learning network that enhances domain generalization by pre-training images with rich semantic knowledge, aligning features from different modalities into a shared space, and fine-tuning with a realistic dataset to adapt to real-world data distributions for superior performance of ViTs has been developed [24]. While ViTs have demonstrated remarkable success in domains like object recognition and medical imaging, with the improvements over time, their application to infectious disease detection remains underexplored. This paper addresses this gap by investigating ViTs for Mpox detection via medical imaging, an area traditionally dominated by CNNs.

The objective of this study is to investigate how ViTs can be leveraged for the detection of Mpox from imaging data, such as skin lesions or other visible symptoms. By comparing the performance of ViTs with conventional CNNs, the goal is to provide a comprehensive analysis of whether ViTs offer superior performance in the context of Mpox detection. Moreover, this paper also evaluates the potential advantages of ViTs, such as their scalability, their ability to generalize across different imaging modalities, and their potential for faster adaptation to emerging variants of diseases such as Mpox. Furthermore, this paper examines the broader implications of ViTs for infectious disease diagnosis, particularly in terms of their ability to revolutionize medical imaging workflows. By reducing the reliance on handcrafted methods for feature extraction and potentially offering improved interpretability and efficiency, ViTs could play a central role in the future image-based diagnosis of infectious diseases. Therefore, this paper not only contributes to the understanding of ViTs in the specific case of Mpox but also paves the way for their broader application in healthcare, where rapid and accurate disease detection is critical.

The rest of this paper is organized into the following sections. Section 2 depicts related works, Section 3 describes the materials and methods, Section 4 presents the results, Section 5 discusses the results, and Section 6 provides the conclusion.

## 2. Related Works

The evolution of deep learning architectures has played a crucial role in improving the performance of models applied to Mpox images [25]. CNNs have been the foundational architecture in this domain, owing to their strong ability to recognize spatial hierarchies in visual data [12]. CNNs operate by applying a series of convolutional filters to an image and effectively detecting edges, textures, and other features that are critical for distinguishing different types of lesions [26]. However, CNNs, although powerful, are limited by their local receptive fields and have difficulty capturing the global context that can be vital for the accurate diagnosis of complex skin conditions such as Mpox [27]. To overcome these limitations, ViTs have emerged as novel architectures for image analysis [16,28]. ViTs, originally developed for natural language processing, adapt the transformer model to visual tasks by treating image patches as sequences of tokens, enabling the capture of long-range dependencies and global information. This shift from local to global processing makes ViTs particularly suitable for analyzing Mpox images, where the distribution of visual features across the entire image is important for accurate diagnosis [29].

Given the relative scarcity of annotated Mpox image datasets, the concept of transfer learning has become an essential part of the development of deep-learning models [30]. In transfer learning, a neural network is first trained on a large dataset, usually unrelated to the target task, and then fine-tuned on a smaller task-specific dataset [31,32]. This approach allows models to leverage vast amounts of data and learned features from broader domains, such as general skin disease datasets, and apply them to Mpox diagnosis with relatively few images. By using pre-trained models, researchers can significantly reduce the amount of data required for training and mitigate the challenges posed by limited data availability [33]. This method has proven successful in various medical image analysis tasks and holds promise for improving the accuracy and reliability of Mpox detection models, even when large datasets are not available. Furthermore, fine-tuning these pre-trained models, specifically for Mpox, allows them to adapt to the unique characteristics of the disease, improving their diagnostic performance. Table 1 summarizes some deep learning methods for Mpox detection from image data.

Despite the progress in deep learning and promising applications in Mpox image analysis, there are still some challenges [13,42]. One of the main challenges is the limited availability of annotated Mpox datasets, which makes the training and validation of deep learning models difficult [43]. This problem of data scarcity is exacerbated by the relatively low incidence of Mpox compared to other diseases, making it difficult to compile large and diverse datasets that can capture the full spectrum of disease manifestations. In addition, the variability in the presentation of Mpox lesions across skin types and disease stages adds further complexity, which may lead to model bias and reduced generalizability [44]. Moreover, the computational resources required to train and deploy advanced models, such as ViTs, are substantial and may not be feasible in all healthcare settings, especially in resource-poor settings where Mpox is most prevalent [45]. Overcoming these challenges will require continued research in data augmentation, model interpretability, and the development of more efficient algorithms tailored to the needs of Mpox diagnosis.

## 3. Materials and Methods

### 3.1. Dataset and Preprocessing

**Figure 1:**
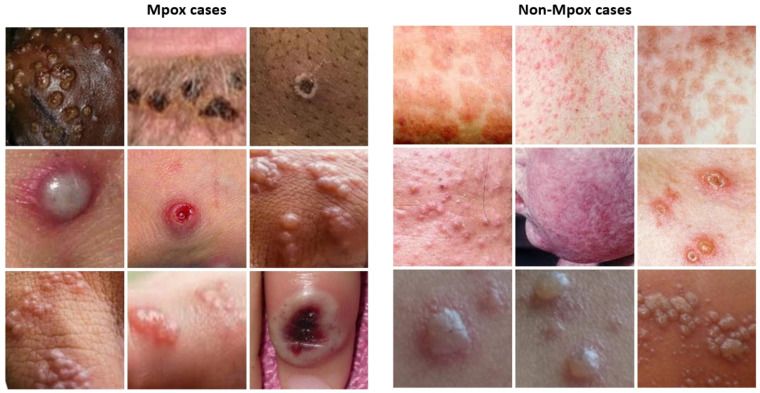
Sample images from the Mpox skin lesion image dataset.

For this study, a dataset of dermatologic images depicting Mpox lesions was obtained from a publicly available dataset (available at https://www.kaggle.com/datasets/nafin59/monkeypox-skin-lesion-dataset [accessed on 19 March 2025]) [46]. The dataset consists of 228 images categorized into two classes: 102 Mpox cases and 126 non-Mpox cases (Measles and Chickenpox because of their resemblance to the Monkeypox rash and pustules in their initial state). The images have a size of 224 × 224 pixels. Sample images from the dataset are shown in Figure 1. Given the relatively small size of the dataset, careful preprocessing and augmentation were critical to enhance model robustness and prevent overfitting. To address this, data augmentation techniques were applied exclusively to the training set, which consisted of 80% of the data, while the remaining 20% was reserved for testing. The augmentation strategies included random rotations, horizontal and vertical flips, and color jittering to simulate a variety of real-world imaging conditions. These transformations aimed to increase the effective diversity of the training data, enabling the model to generalize better across different lighting conditions, orientations, and image artifacts [47].

### 3.2. Vision Transformer Architecture

In this study, a pre-trained ViT model was used to classify Mpox images using a transfer learning approach. The ViT model, which was originally pre-trained on a large-scale ImageNet dataset, was adapted for the specific task of Mpox classification. Vision transformers process images by dividing them into fixed-size patches, embedding them into a sequence of vectors, and then processing the sequence using transformer layers. This method is particularly effective for capturing global dependencies in image data that are critical for accurate classification [48]. In ViTs, each transformer encoder layer contains two key components: a multi-head self-attention mechanism and a position-wise multilayer perceptron (MLP) block. The MLP block typically consists of two fully connected layers with a non-linear activation function (such as GELU) in between. It helps in learning complex transformations of the token representations after the attention operation, contributing significantly to the model’s ability to capture high-level patterns in visual data.

**Figure 2:**
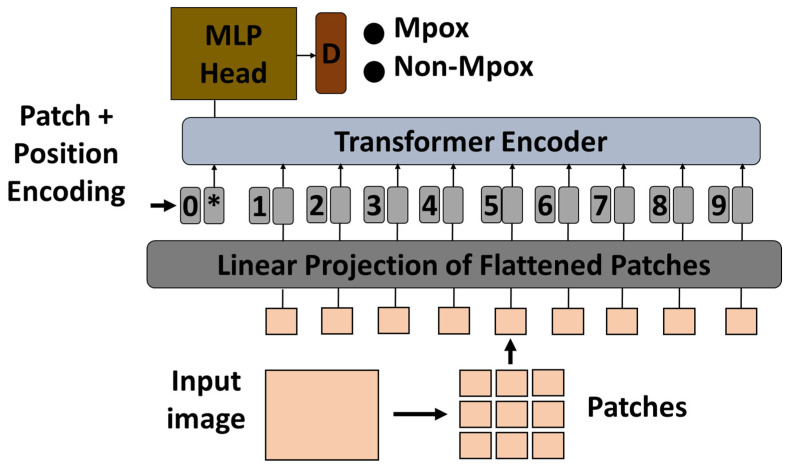
Vision transformer-based transfer learning architecture. MLP, multilayer perceptron; *, extra learnable (class) embedding.

To adapt the pre-trained ViT model to the Mpox classification task, the final classification head was replaced with a new layer corresponding to the number of Mpox classes in the dataset. The original classification head, which was designed for general tasks such as object recognition, was no longer relevant for the specific task of Mpox detection. The new classification head allowed the model to produce results that were targeted at specific categories of Mpox lesions or disease manifestations captured in medical images. After modifying the classification head, the remaining ViT model was fine-tuned to the Mpox dataset to ensure that the transformer layers were adapted to the characteristics of that particular disease. During fine-tuning, the model was re-trained with the new classification head, while the learning rate and other hyperparameters were adjusted to optimize performance. The effectiveness of the fine-tuned ViT model was then evaluated on a held-out test set to measure its accuracy and generalization capabilities in classifying Mpox images. Figure 2 shows the proposed architecture.

### 3.3. Training Procedure

During fine-tuning, the model was re-trained while retaining much of the knowledge that the ViT had acquired from large-scale datasets such as ImageNet. Rather than retraining the model from scratch, fine-tuning leveraged the already trained transformer layers, which already encoded useful general features such as basic shapes, edges, and textures. These layers were further refined to capture Mpox-specific patterns such as the shape, texture, or distribution of the lesions. The fine-tuning was conducted by first freezing some of the earlier layers of the ViT, which were responsible for extracting general features from the input images. The deeper layers, which handle more abstract representations, were left unfrozen and retrained to focus on the unique aspects of Mpox. This allowed the model to retain the benefits of the pretraining while adapting its internal representations to the new task. During fine-tuning, careful attention was paid to adjusting key hyperparameters, such as the learning rate, batch size, and weight decay. A lower learning rate was used to avoid overwriting valuable pre-trained knowledge in the transformer layers and to ensure that the model learns from the Mpox dataset. In tuning the hyperparameters, several experiments were conducted to determine the best combination of these values to ensure that the model converged to the optimal performance for Mpox detection.

The transfer learning process included the following.

- Pre-trained ViT selection: a ViT model pre-trained on a large dataset, such as ImageNet, was selected as the starting point for the adaptation;
- Modification of the classification head: the final classification head was replaced by a new layer corresponding to the number of Mpox classes in the target dataset;
- Freezing the layers: the initial layers of the ViT, responsible for extracting basic image features, were frozen, whereas the final layer was trained to focus on Mpox-specific features;
- Hyperparameter tuning: critical hyperparameters such as the learning rate were carefully adjusted to ensure that the model learned from the Mpox dataset without losing the knowledge gained during pretraining;
- Model training and fine-tuning: The model was fine-tuned using the Mpox dataset, so that deeper transformer layers could learn disease-specific patterns. This step involves multiple iterations to refine the performance of the model;
- Performance evaluation: After fine-tuning, the performance of the model was evaluated using metrics such as accuracy, precision, recall, and F1-score.

The ViT model was trained using the Adam optimizer, which is particularly effective for handling large-scale datasets and high-dimensional parameter spaces. The learning rate was initially set to 0.0001 and dynamically adjusted using learning-rate annealing based on validation loss progression. A batch size of 32 was selected to balance GPU memory consumption and training efficiency. The model was trained over 50 epochs, a value empirically determined through iterative testing. Cross-entropy loss was used for classification, with dropout (rate of 0.1) incorporated to prevent overfitting. Key architecture-specific parameters such as a patch size of 16 × 16, embedding dimension of 768, 12 transformer layers, and 12 attention heads were maintained, as per standard ViT configurations. All experiments were conducted on a high-performance computing cluster equipped with NVIDIA GPUs (NVIDIA Corporation, Santa Clara, CA, USA) to ensure efficient and reproducible training.

### 3.4. Evaluation Metrics

The trained vision transformer model was evaluated using a separate test set that was not used during training or validation. Key evaluation metrics included accuracy, precision, recall (sensitivity), F1-Score, and area under the receiver operating characteristic curve (AUC) [49].

In addition to conventional machine-learning evaluation metrics, a transferability metric was used in this study to evaluate the effectiveness of vision-transfer-based transfer learning for classifying Mpox images. In machine learning, a transferability measure evaluates how effectively knowledge learned from one task, typically through a pre-trained model, can be transferred to a different but related task [50]. This quantifies the efficiency of transferring learned features, representations, or weights from a source task to a target task as part of transfer learning. Transferability is usually higher when the source and target tasks are similar, because the measure captures how well the features learned from the source task can be generalized to the new task. One of the main goals of transferability is to improve performance on the target task by leveraging the knowledge from the pre-trained model. The transferability measure helps determine how much improvement, or potential degradation, occurs compared to training the model from scratch. In addition, the reusability of the learned features in a new context is assessed by examining whether the pre-trained representations are effective or whether significant fine-tuning is necessary [51]. In cases where the source and target tasks originate from different domains, the transferability measure reflects the extent to which the model can handle domain shifts and still produce useful results for the target task. In this study, the hypothesis margin was used to assess the transferability in this study.

The hypothesis margin (HM), which measures the difference between the model’s confidence in predicting the correct class and the highest confidence among incorrect classes, has been shown to be insightful for evaluating transferability within transfer-learning paradigms [52]. When assessing the confidence and generalization of the model across domains, a wider margin indicates stronger adaptation and generalization capabilities, which are essential for robust performance on new tasks. In addition, changes in the margin between the source and target domains can signal shifts in the domain, indicating the need for domain adaptation strategies. Thus, the hypothesis margin serves as a concise yet comprehensive metric for gauging the efficacy of transfer learning models in seamlessly transferring knowledge across disparate domains [53]. The practical advantage of log-expected empirical prediction (LEEP) [54] and an H-score [55] lies in their minimal data requirements, as they rely solely on the model and the target dataset for calculation and do not require any data from the source dataset. However, this simplicity has a theoretical disadvantage, as it is difficult to identify the specific characteristics of the source dataset that contribute to its pretraining efficiency. It is necessary to use several measures of statistical characteristics of the dataset characteristics to allow for a clear interpretation. Hypothesis margin serves as one such measure that quantifies the margins between sets of points with different labels. This concept is used extensively in feature selection and as part of the loss functions in machine learning.

For a single point x, the HM is given by (1), where the nearest point to x, but in a different class from x is nearmiss(x) and the nearest point to x that is in the same class is nearhit(x).

(1) $$M\left(x\right)=\frac{1}{2}\left(\left\|x-nearmiss(x)\right\|-\left\|x-nearhit(x)\right\|\right)$$

The average hypothesis margin (2) was used to calculate multiple points in our data. The margin between learned features extracted from points in the source dataset and learned features extracted from points in the target dataset was evaluated by using the feature extractor ∅ from a model pre-trained on the source dataset using the average hypothesis margin $\bar{M}\left(X_{t},X_{s}\;\right)$.

(2) $$\bar{M}\left(X_{t},X_{s}\;\right)=\frac{1}{2(\left|X_{t}\right|+\left|X_{s}\right|)}\sum_{x\in X_{t}\cup X_{s}}\left\|\emptyset \left(x\right)-\emptyset (nearmiss(x))\right\|-\left\|\emptyset -\emptyset (nearhit\left(x\right))\right\|$$

### 3.5. Experimental Settings

To thoroughly evaluate the effectiveness of the proposed method, four distinct experiments were conducted. Each experiment tested a different aspect of the ViT-based transfer learning approach in the context of Mpox recognition to ensure a comprehensive evaluation of its performance and feature transferability.

The first experiment was designed to compare the ViT-based and the conventional CNN-based transfer learning approaches. This comparison was made using established transfer learning metrics such as accuracy, precision, recall, and F1-score. The goal was to determine whether the ViT-based model outperforms the widely used CNN models, which are usually the preferred architectures for image classification tasks, including medical image analysis. This experiment was crucial to establish a baseline performance comparison between the two architectures when adapted using transfer learning for the specific task of Mpox detection.

The second experiment aimed to assess the effectiveness of the proposed ViT-based transfer learning approach compared to models trained from scratch. In this setup, the pre-trained ViT model was fine-tuned on the Mpox dataset, and its performance was compared with that of models initialized with random weights and trained entirely on the same dataset. This experiment helped provide insight into the advantages of transfer learning over training from scratch, especially in cases where the dataset for the target task is relatively small, as is often the case with medical datasets. This shows that pre-trained models, with their rich feature representations learned from large datasets, offer a significant advantage in terms of training efficiency and final performance.

The third experiment focused on evaluating feature transferability between the ViT- and CNN-based transfer learning approaches. The goal of this experiment was to measure how well the features learned by the pre-trained ViT model were transferred to the new Mpox detection task compared to the features learned by pre-trained CNN models. Transferability was evaluated by examining the model’s transferability using the HM to determine how effectively the ViT and CNN models captured relevant Mpox-specific patterns during the fine-tuning process. This experiment provides a deeper insight into the advantages of transformer-based architectures for feature extraction and transfer, highlighting their ability to capture global dependencies in an image.

The fourth experiment introduced a qualitative evaluation using gradient-weighted class activation mapping (Grad-CAM) to visually interpret the effectiveness of the proposed ViT-based transfer learning approach. Grad-CAM was used to create heat maps that highlighted the regions of the input images that the model focused on when making its predictions. This visual analysis was critical for understanding whether the ViT-based model considered the most relevant regions of the Mpox images, such as lesions or affected areas, compared to the CNN-based models.

## 4. Results

Four experiments were conducted to evaluate the effectiveness of the proposed method. The first experiment was conducted to compare the ViT-based transfer learning approach with the CNN-based TL approach using conventional transfer learning metrics. The second experiment was conducted to evaluate the effectiveness of the proposed TL algorithm compared to models trained from scratch. The third experiment evaluated the effectiveness of feature transferability of the ViT-based TL approach compared to the CNN-based TL approach. The fourth experiment was performed to visually evaluate the effectiveness of the proposed ViT-based TL approach using Grad-CAM outputs. Taken together, these four experiments provided a comprehensive evaluation of the proposed ViT-based transfer learning approach and compared it to CNN-based models in terms of conventional metrics, feature transferability, training efficiency, and interpretability. The results of these experiments highlight the potential of ViT models to significantly improve the performance in Mpox detection and make a strong case for their use in the classification of medical images where transfer learning is required.

**Figure 3:**
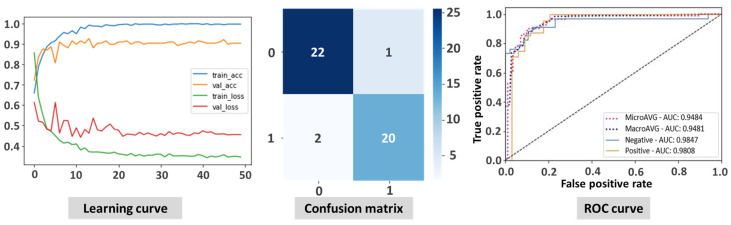
Illustration of the results of vision transformer-based transfer learning displaying the learning curve, confusion matrix, and ROC curve.

The ViT model showed superior performance in the classification of Mpox images compared to state-of-the-art CNN-based models. The ViT model proved to be highly effective in classifying Mpox images, as illustrated by the results presented in Figure 3. The learning curve shows the rapid and stable convergence of the model, indicating an efficient learning process and the ability to generalize well from the training data to unseen test data. The confusion matrix illustrates the accuracy of ViT, with a high number of correct classifications and very few cases of misclassifications, which are crucial for minimizing errors in the clinical setting. In addition, the ROC curve illustrates the strong discriminatory power of the model with an AUC of 0.948, reflecting its exceptional ability to distinguish between Mpox and non-Mpox cases. Overall, these figures demonstrate the robustness and reliability of the ViT model in accurately identifying Mpox cases.

ViT achieved an accuracy of 0.942, which was significantly higher than that of ResNet-50 (0.895) and EfficientNet (0.917) with *p*-value < 0.05 in all cases, Table 2. This superior performance is likely due to the ability of ViT to capture long-range dependencies and global context in images, which are critical for accurately identifying the complex visual patterns associated with Mpox. The ViT model also exhibits strong precision and recall, with values of 0.936 and 0.953, respectively, indicating a low rate of false positives and a high rate of true positives, making the model highly reliable for clinical use. The F1-score of 0.944 further underscores the robustness of the model by balancing the precision and recall. In addition, ViT achieved an AUC of 0.948, reflecting its excellent ability to distinguish between Mpox-positive and Mpox-negative cases. In contrast, ResNet-50 and EfficientNet performed well but had limitations in AUC, F1-score, precision, recall, and overall accuracy, emphasizing the advantages of the ViT model.

The comparison between transfer learning and training from scratch highlighted the significant advantages of transfer learning in all models tested, with ViT showing the most striking improvement, Table 2. In transfer learning, ViT achieved an impressive accuracy of 0.942, along with high precision (0.936), recall (0.953), F1-score (0.944), and AUC (0.948), clearly outperforming the other models. These results demonstrate how well ViT leverages pretrained weights to extract useful features from large, generalized datasets, allowing it to achieve state-of-the-art performance on specific tasks. In contrast, ViT’s performance drops significantly when trained from scratch, with an accuracy of only 0.691, a precision of 0.703, and a recall of 0.71, indicating that it requires considerably more data and training time to achieve a comparable level of performance. This stark contrast illustrates the challenges of training complex models, such as ViT, without the benefits of transfer learning. For all models, transfer learning consistently led to better performance. For example, both ResNet50 and EfficientNetB2 benefit from transfer learning, although not to the same extent as ViT. ResNet50 achieved an accuracy of 0.895, and EfficientNetB2 an accuracy of 0.917 when pretrained, whereas both models showed much lower performance when trained from scratch. These results suggest that while simpler models such as ResNet50 and EfficientNetB2 can perform decently from scratch, the more sophisticated and computationally intensive ViT relies heavily on pretrained models to achieve optimal results. Overall, this comparison emphasizes that transfer learning, especially for large and complex architectures, such as ViT, provides a significant performance boost, making it the preferred approach for achieving high accuracy and efficiency in computer-vision tasks.

**Figure 4:**
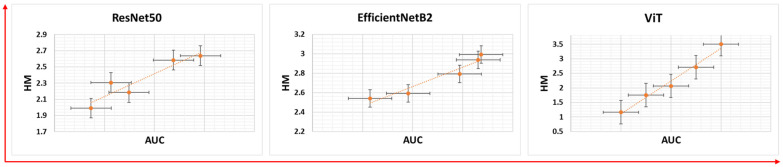
Transferability trends for different models drawn against their respective AUC values.

The transferability of features for each model was evaluated using the HM measure. Each model was run five times, each with different distribution of training and test data, to assess the transferability patterns. The results showed that higher AUC values were associated with higher HM values (Figure 4), indicating a positive correlation between th AUC and HM, as evidenced by the Pearson correlation coefficients in Table 3. Looking at the HM values of different architectures, ViT shows consistently higher HM values (Figure 4) and a stronger correlation between the AUC and HM than EfficientNetB2 and ResNet50.

**Figure 5:**
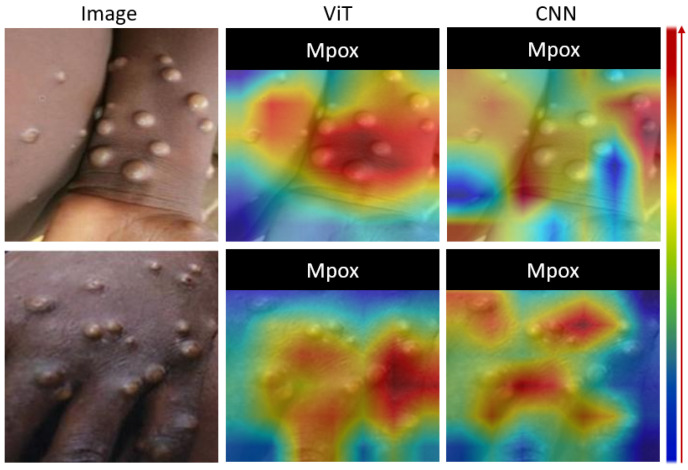
Grad-CAM output comparison of ViT- and CNN-based transfer learning for Mpox images.

Figure 5 shows a visual analysis of the proposed model using Grad-CAM. Grad-CAM analysis provides a visual interpretation of the focal areas of deep learning models in identifying Mpox lesions. The left column shows the original images of the skin with Mpox. The right column contains the corresponding Grad-CAM heatmaps, with the color gradients indicating the model’s attention. The red highlights represent the most important areas for classification, where the model strongly associates the features with Mpox, yellow to green zones indicate moderate attention, and blue signifies regions with little or no relevance for the model’s decision. Of note, the red regions closely align with the raised pustules in each image, indicating that the model accurately identifies these lesions as primary markers of Mpox. This visualization demonstrates the model’s ability to focus on relevant clinical features, particularly the shape, size, and texture of the pustules, which increases confidence in the interpretability and reliability of its predictions.

We have also performed a comparison with the state-of-the-art ViT methods that were developed in the past two years for Mpox detection, Table 4. Direct comparison of our method with existing approaches for Mpox detection is challenging due to differences in the training and testing settings, and in some cases the datasets. However, the results indicate that our method performs comparably or better than existing methods. Unlike Aloraini et al. [38], who reported higher performance by applying extensive data augmentation to both the training and test sets, our study adhered to a more realistic evaluation approach. We applied a reasonable amount of augmentation solely to the training data, while preserving the original, unaltered images for testing to better reflect real-world deployment conditions.

## 5. Discussion

The results of this study have shown that the ViT model is a highly effective tool for classifying Mpox images and shows better performance than CNNs. This success can be attributed to several key advantages of ViTs over CNNs. Unlike CNNs, which extract features hierarchically through convolutional layers, ViTs process an entire image as a sequence of patches, allowing them to capture both local and global features. This approach allows ViTs to recognize subtle and distant patterns that are critical for accurate detection of Mpox. The model’s capacity to discern associations between remote areas of a picture is especially advantageous in medical imaging, where the context and distribution of visual features are critical to diagnosis. In addition, ViTs are highly scalable and efficiently manage large images and datasets, which is a common challenge in medical imaging due to varying image resolutions and qualities.

Furthermore, the effectiveness of the ViT model through transfer learning shows that it is also suitable for new or emerging diseases for which large disease-specific datasets are not available. In situations such as Mpox analysis, where there are limited labeled data, transfer learning allows the ViT model to leverage knowledge from vast pretrained datasets, making it highly adaptable to unfamiliar diseases. This capability is critical for rapid response in public health emergencies, as it allows healthcare providers to deploy advanced AI tools for diagnosis without having to wait for extensive data to be collected and labeled, a process that can be time-consuming and resource-intensive. By using pretrained ViT models, healthcare systems can apply these tools to analyze cases of emerging diseases with high accuracy, even when data are scarce or incomplete. This approach is not only cost-effective, but also scalable, as the same pretrained model can be fine-tuned to recognize patterns in different new diseases as they emerge. For example, if a new virus or skin disease breaks out in the future, a ViT model could be quickly adapted by retraining a small number of cases. This would help doctors to identify the visual patterns of the disease and understand its characteristic features without the need for a vast dataset. This adaptability makes the ViT model a powerful tool in areas such as epidemiology and infectious disease control, where timely diagnosis and containment are critical. In addition, ViT’s flexibility in handling new diseases has expanded its applicability in global healthcare, particularly in low-resource settings. In regions with limited access to extensive diagnostic databases, ViT models can be fine-tuned on small datasets to enable accurate diagnosis, thus reducing the obstacles to the implementation of AI-assisted healthcare in these areas. This is particularly valuable for diseases that have not yet been extensively studied. Here AI can help identify early warning signs and assist in monitoring disease progression. By improving the accessibility and effectiveness of diagnostic AI tools for new diseases, ViT models can play a pivotal role in strengthening global health resilience and ensuring timely and accurate responses to evolving health threats.

Although the effectiveness of the ViT model through transfer learning highlights its promise for new or emerging diseases with limited datasets, its broader application in clinical practice still faces challenges related to computational costs and the need for large amounts of annotated data. ViTs are computationally intensive as they rely on attention mechanisms and their ability to process high-dimensional data, which may be a significant obstacle in resource-poor settings, such as smaller clinics or in regions with limited access to high-performance computing resources. Compared to the best performing CNN, EfficientNetB2, ViT is computationally expensive having 80 M trainable parameters (against 5 M for EfficientNetB2), requiring longer training time of 3 h over 50 epochs (against 2 h for EfficentNetB2), and having a GFLOPs value of 17 (against 2 for EfficientNetB2). Although transfer learning allows ViTs to perform well with smaller datasets, achieving optimal performance often requires extensive annotated data for fine-tuning, which can be time-consuming and costly, especially for new or rare diseases. The availability of large-scale labeled medical images is often limited due to the high cost and complexity of obtaining expert annotation, privacy concerns, and regulatory restrictions on data sharing. This can affect the generalizability of the model, even when pretrained weights are used. Nevertheless, solutions, such as developing more computationally efficient versions of ViT models, using self-supervised or semi-supervised learning techniques, and using federated learning for privacy-preserving data sharing, could mitigate some of these challenges. These strategies could help overcome the barriers to the use of ViT models in a variety of healthcare settings and make them more accessible and scalable for diagnosing emerging diseases, such as Mpox, where rapid adaptation and deployment are crucial for timely intervention and containment.

This study has its limitations. First, it relies on a single Mpox lesion image dataset, which may limit the model’s generalizability to different populations or real-world conditions with varying image qualities or lesion types. Moreover, the study lacks external validation on independent datasets, which is crucial for confirming the model’s robustness. While a transferability measure was used to assess feature transfer, its practical relevance and standardization for Mpox diagnosis are not fully established. Although Grad-CAM visualizations were used to support model interpretability, the approach’s reliability in complex or atypical cases was not thoroughly explored. Furthermore, the study primarily compares ViTs to CNN-based models and ViTs trained from scratch, without evaluating other emerging or hybrid architectures that could potentially offer better performance. These factors suggest that further research is needed to enhance the model’s robustness, data diversity, and real-world applicability.

Building on the insights and limitations of this study, several prospects emerge. First, expanding the dataset to include a more diverse range of Mpox images—such as those from different geographic regions, patient demographics, and lesion variations—could enhance the model’s generalizability and real-world applicability. Additionally, incorporating external validation using independent datasets from multiple sources would provide a more robust assessment of the model’s performance and its ability to handle diverse clinical conditions. Exploring alternative advanced architectures, including hybrid models that combine the strengths of ViTs and CNNs, could further improve diagnostic accuracy and robustness. Further investigation into the model interpretability, beyond Grad-CAM visualizations, is essential for understanding its decision-making in complex or atypical cases. Finally, addressing ethical concerns related to data privacy, bias, and transparency will be crucial for the wider adoption of AI-based diagnostic tools in healthcare. These advancements could pave the way for more effective, scalable, and clinically reliable AI-driven diagnostic solutions for Mpox and other infectious diseases.

This study has several practical implications. First, the ViT-based model’s superior accuracy offers the potential for rapid and precise Mpox detection, improving early diagnosis and treatment, particularly in resource-limited settings where traditional methods are slower and more equipment-dependent. Second, AI-driven diagnostic tools could enable scalable and accessible screening for Mpox, especially in non-endemic regions, helping to broaden access to high-quality diagnostics and strengthen global surveillance efforts for diseases such as measles. Finally, the application of transfer learning in medical imaging, as demonstrated in this study, provides a cost-effective pathway for developing robust diagnostic models with limited data, offering potential for quick adaptation to other infectious diseases and health crises.

In general, despite the challenges, the exceptional performance of the ViT model in classifying Mpox images indicates its potential for a wide range of diagnostic applications. Its ability to manage complex visual patterns and provide interpretability makes it a promising tool for various medical imaging tasks beyond Mpox. Future research should focus on validating the generalizability of ViTs to different imaging modalities and disease types to fully exploit its potential to improve diagnostic accuracy and clinical decision-making. The success of the ViT model in this study marks a significant step forward and demonstrates that vision transformers can play a pivotal role in the future of medical image analysis.

## 6. Conclusions

This study demonstrates that vision transformers (ViTs) offer a promising approach for Mpox detection in medical imaging, showing improved performance over conventional CNN-based models within the scope of the available dataset, although further validation on external data is needed to confirm their diagnostic reliability and generalizability. By leveraging transfer learning, the ViT model achieved high accuracy, even with limited training data, underscoring the value of pre-trained models in resource-poor contexts and emerging diseases. Furthermore, the interpretability afforded by ViT’s Grad-CAM visualizations reinforces its clinical relevance, as the model accurately captures disease-relevant features such as lesion shapes and textures. Despite some challenges, such as the high computational cost and limited annotated datasets, ViTs represent a scalable, adaptable solution for the rapid diagnosis of infectious diseases and have tremendous potential to improve disease management in global healthcare facilities. Furthermore, this study is limited by its limited exploration of interpretability in complex cases and potential constraints in generalizability to diverse real-world settings. Future research should focus on optimizing ViTs for medical applications, particularly to reduce their computational requirements and improve their performance on smaller datasets. As the field of AI in healthcare continues to evolve, ViTs can play a central role in next-generation diagnostic tools and contribute to more accurate, efficient, and accessible healthcare solutions.

## Figures and Tables

**Table 1:** Deep learning research for analyzing Mpox images.

Paper	Method	Neural Network	Year
Asif et al. [34]	Ensemble based on metaheuristics optimization	CNN	2023
Ahsan et al. [28]	Transfer learning based on federated learning	ViT	2024
Nayak et al. [29]	Transfer learning	CNN	2023
Kularathne et al. [35]	Synthetic data generation-based transfer learning	ViT	
Sun et al. [36]	A two-branch depth separable convolution residual squeeze and excitation module	CNN	2024
Kundu et al. [37]	Generative adversarial network (GAN)-augmented dataset-based federated learning	GAN	2024
Aloraini [38]	Transfer learning	ViT	2024
Oztel [39]	Transfer learning	ViT	2024
Vuran et al. [40]	Self-supervised learning, self-distillation, and shifted window techniques	Transformer	2025
Cao et al. [41]	Mask, inpainting, and measure (MIM)	GAN	2025

**Table 2:** Experimental results of the proposed method and comparison performed.

Method	Model	Accuracy	Precision	Recall	F1-Score	AUC	Training Time (h)
Transfer learning	ResNet50	0.895	0.882	0.901	0.891	0.893	0.37
	EfficientNetB2	0.917	0.905	0.911	0.912	0.911	0.42
	ViT	0.942	0.936	0.953	0.944	0.948	0.61
Training from scratch	ResNet50	0.683	0.667	0.675	0.662	0.668	0.44
	EfficientNetB2	0.674	0.663	0.658	0.653	0.662	0.61
	ViT	0.691	0.703	0.710	0.698	0.709	0.94

**Table 3:** Comparison of Pearson correlation coefficients between HM transferability measures and AUC values.

Model	AUC	HM Pearson Correlation Coefficient Values
ResNet50	0.8926 ± 0.0012	0.826
EfficientNetB2	0.9142 ± 0.0018	0.839
ViT	0.9499 ± 0.0011	0.872

**Table 4:** Comparison of the proposed method with state-of-the-art ViT methods for Mpox detection.

Study	Accuracy	Year
Ahsan et al. [28]	89.00%	2024
Aloraini [38]	94.69%	2023
Oztel [39]	81.91%	2024
Ours	94.20%	2025

## Data Availability

In this study, we used a publicly available Mpox image dataset from the Monkeypox Skin Lesion Dataset (https://www.kaggle.com/datasets/nafin59/monkeypox-skin-lesion-dataset [accessed on 19 March 2025]).
